# Novel *ERCC2* variant in trichothiodystrophy infant: the first case report in China

**DOI:** 10.1186/s12887-021-02585-4

**Published:** 2021-03-12

**Authors:** Jian-Dong Chen, Wei-Dong Liao, Ling-Ying Wen, Rong-Hua Zhong

**Affiliations:** Department of Neonatology, Longyan First Affiliated Hospital of Fujian Medical University, No.105, Jiyi North Road, Xinluo District, Longyan, 364000 Fujian China

**Keywords:** *ERCC2*, Trichothiodystrophy, Brittle hair, Genodermatoses, Novel variant

## Abstract

**Background:**

Trichothiodystrophy (TTD) is a rare, autosomal recessive, multisystem disorder most commonly caused by variants in *ERCC2*.

**Case presentation:**

Here, we describe the first Chinese patient with a novel variant in *ERCC2*. A male infant, who was born to a healthy non-consanguineous couple, exhibited brittle hair, hair loss ichthyosis, eczema, retinal pigmentation and hypospadias. He carried a novel heterozygous *ERCC2* variant. The maternal variant (c.2191-18_2213del) is a previous described genomic deletion that affects the splicing of intron 22. The paternal variant (c.1666-1G > A), that occurs in the splice site of intron 17 and likely alters *ERCC2* gene function through aberrant splicing, has not been reported previously.

**Conclusions:**

Our case reported a novel pathogenic variant in *ERCC2*, which expanded the known genetic variants associated with TTD.

**Supplementary Information:**

The online version contains supplementary material available at 10.1186/s12887-021-02585-4.

## Background

Trichothiodystrophy (TTD) is a rare, autosomal recessive, multisystem disorder, which is characterized by sulfur-deficient brittle hair, ichthyosis, cutaneous photosensitivity, mental and physical retardation [1]. Usually, TTD is caused by the defects in transcription and nucleotide excision repair due to variants in DNA transcription-repair genes (*ERCC2*, *ERCC3* and *GTF2H5*), which encode the distinct subunits of transcription/repair factor IIH (TFIIH) [2]. The majority of TTD cases involved variants in the *ERCC2* gene and presented a remarkable phenotypic heterogeneity [3]. In Asian countries, only a few cases were reported due to the low incidence [4]. Here, we report the first infant case with TTD from China. The case was caused by a compound heterozygous *ERCC2* variant, in which maternal variant (c.2191-18_2213del) has been described previously, but the paternal variant (c.1666-1G > A) is novel.

## Case presentation

A male infant was born at 36 weeks’ gestation by caesarean section due to fetal distress. The parents were non-consanguineous and the family history noncontributory. The postnatal period of the newborn was complicated by polypnea. At birth, his weight and body temperature were 1.71 kg and 35.4 °C respectively, thus he was admitted to hospital for the first time due to low body weight and premature birth. On physical examination, his entire body was covered by the dry, parchment-like skin, without pigmentation. He presented abnormal genital appearance, including flat glans and hypospadias, and his urinary meatus was located in frenulum of prepuce. His arterial blood gas test, blood biochemistry, complete blood count, urine analysis, screening for genetic metabolic diseases by tandem mass spectrometry and cranial color Doppler ultrasound revealed no obvious abnormality. Ultrasound cardiogram showed an atrial septal defect and patent foramen ovale.

On the second day of hospitalization, he underwent phototherapy due to hyperbilirubinemia. Based on these clinical manifestations on skin and genital, a tentative diagnosis of TTD was made. For further confirmation, peripheral blood was collected for whole exome sequencing (WES) using Illumina Hiseq (Illumina, USA, Supplementary Table 1 and Table 2). During his 14 days of hospitalization, he was administrated with amoxicillin/sulbactam due to neonatal infectious pneumonia and other symptomatic treatments. At 14 days of birth, his overall clinical condition improved (weight 2.0 kg) without polypnea and xanthochromia, and he was discharged. According to his parents’ description, infant presented with irregular hair loss after discharge.

**Fig. 1:**
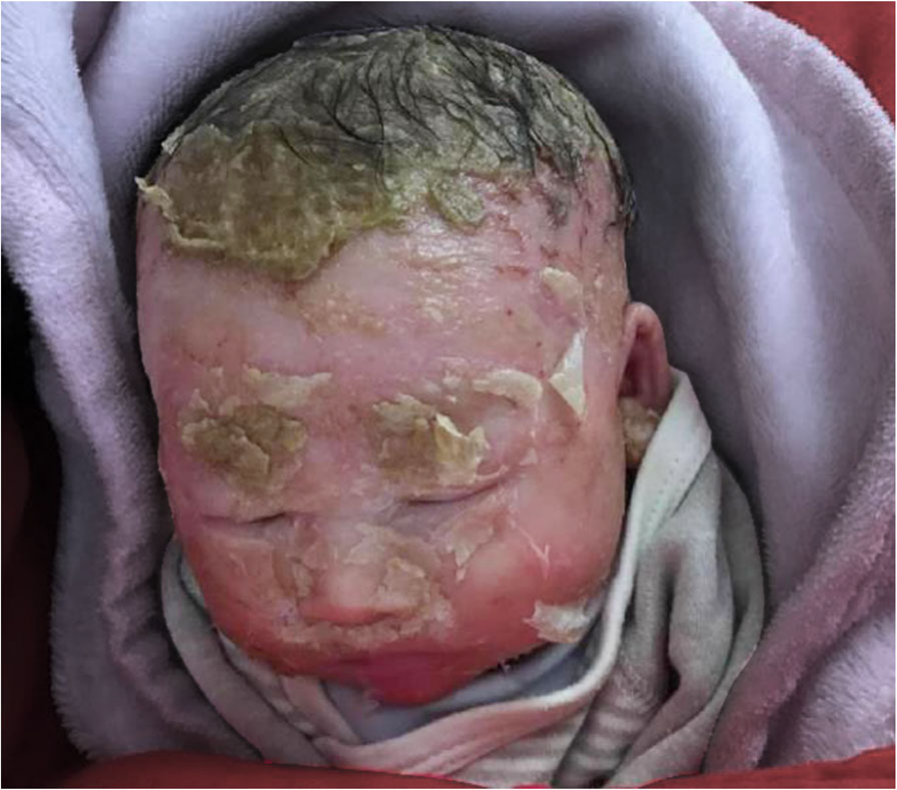
Clinical features of the patient. He has parchment-like skin, extensive eczema, thin, brittle, and sparse hair

At 36 days of birth, he presented to hospital due to neonatal infectious pneumonia. On physical examination, patient continued to demonstrate brittle hair, irregular hair loss (Fig. 1), ichthyosis, and abnormal genital appearance. Bilateral testicles were descended. Besides, infant presented with extensive eczema on the parietal region of his head skin (Fig. 1). At admission, chest X-ray showed infectious pneumonia. After 12 days treatment, due to unsatisfactory curative effect, thoracic spiral computed tomography reexamination was performed, showing multiple pneumonia episodes. Ocular examination revealed whitening of the retina in zone II area and retinal pigmentation in zone III area. During his 1 month of hospitalization, he was administrated with oxygen inhalation, anti-biotic drugs (amoxicillin/sulbactam or meropenem), dopamine, nebulizer therapy and other symptomatic treatments. On the fifth day of admission, he was transfused with red blood cells to correct anemia. The infant passed away at the age of 8 months after being discharged from hospital due most likely to disease aggravation.

**Fig. 2:**
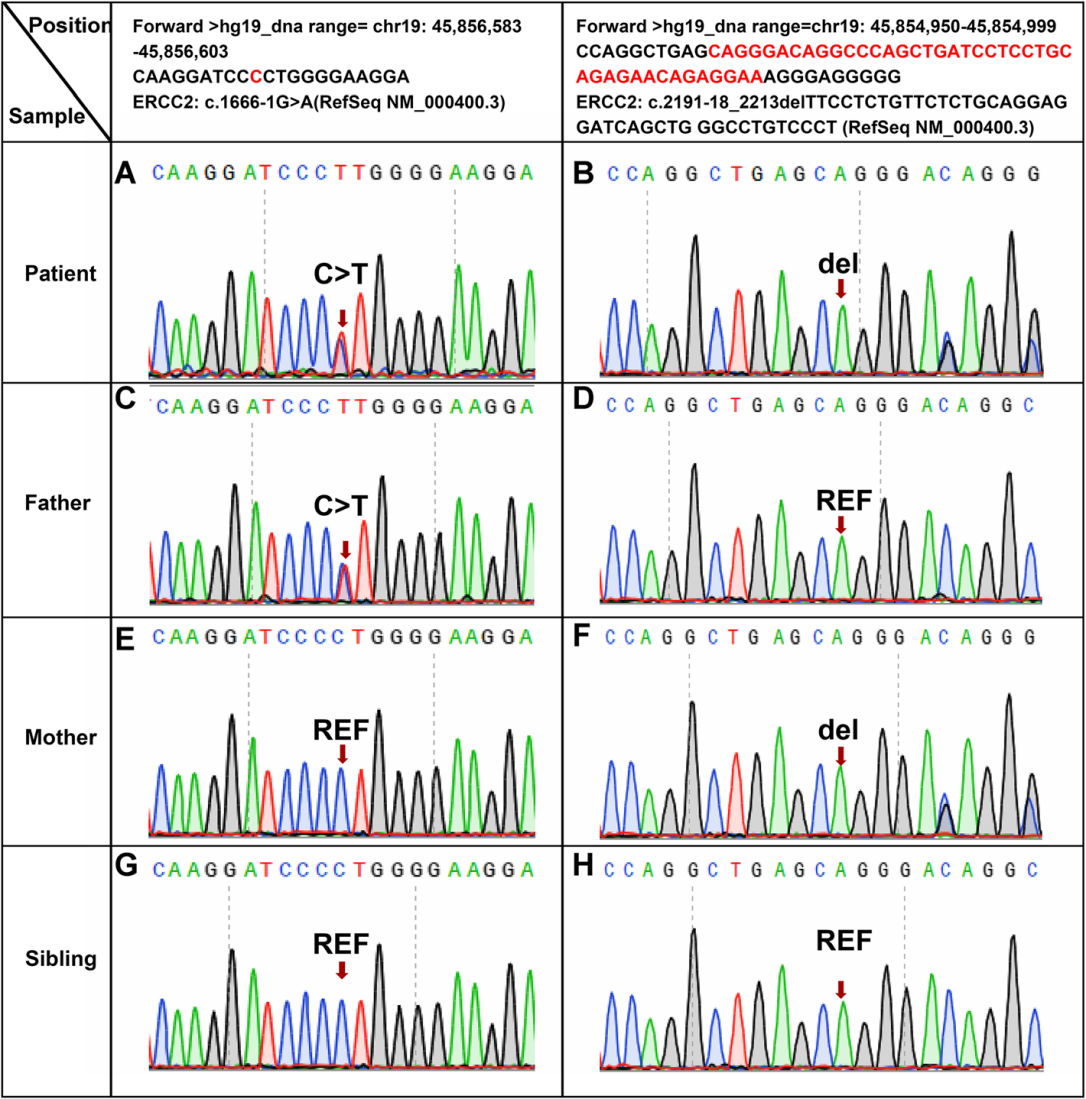
Sequence chromatograms of *ERCC2* gene in the patient and his kinsfolk. REF, reference

He was the second child of healthy non-consanguineous Chinese parents. His male 2-years old sibling (same parents) was healthy without any problems. During the parents’ second pregnancy, delivery was by induction of labour due to fetal distress. During this third pregnancy, the antenatal period had been uneventful. Down syndrome had been ruled out via amniocentesis. Postnatal genetic analysis by using WES revealed a compound heterozygous variant in *ERCC2* gene (Fig. 2a and b). One variant, c.1666-1G > A (RefSeq NM_000400.3), is paternal variant; the other, c.2191-18_2213del (RefSeq NM_000400.3, p.Glu731fs), is maternal variant. Singleton WES confirmed the heterozygosity in both parents (Fig. 2c-f). The variants in *ERCC2* gene were not found in his sibling (Fig. 2g and h). Further in silico analysis by Human Splicing Finder version 3.0 (HSF3.0) demonstrated that the paternal variant most probably affects splicing, with the variation in the wild-type sequence estimated to be 86.54, and variation for the mutated sequence being 58.67 (ΔCV: − 32.2%). According to clinical manifestations and genetic analysis, case was diagnosed with TTD.

## Discussion and conclusions

In this case report, we describe an infant with compound heterozygous variants in *ERCC2* gene. The patient clinically exhibited typical TTD phenotypes, manifesting as brittle hair, hair loss, ichthyosis, eczema, retinal pigmentation, cardiac disorders and gonadal dysgenesis. Based on these multisystem disorders and genetic analysis, patient was diagnosed with TTD.

TTD is recessive condition involving multiple organs and body systems, presenting with a wide spectrum of cutaneous, immune, neurologic, and developmental abnormalities [5]. Several previous review have indicated that frequent features of TTD were abnormalities in hair, nervous system, skin and eye [6, 7]. In this report, patient presented with several common features, such as brittle hair, ichthyosis and retinal pigmentation. Sulfur-deficient brittle hair usually presents with the typical “tiger-tail banding” pattern with alternating light and dark bands under polarizing light microscopy [8]. However, the hair of individuals with TTD may not manifest the “tiger-tail banding” pattern until 3 months old [9]. Thus, we did not observe this specific pattern in our case. Additionally, our patient was born preterm (36 weeks) and small for gestational age, which was in accordance with the previous reports [1, 10]. As Moslehi et al. described previously, TTD-related genes are important for normal placental development, in which there is increased the risks of gestational complications (preterm delivery, small for gestational age, etc.) [11]. A wide variety of ocular abnormalities including infantile cataracts, retinal pigmentation, refractive error, and nystagmus have been reported previously [12]. Here, we observed the retinal pigmentation in this case. Except for these common features, we observed several less common abnormalities, including cardiac disorders and hypospadias. All of these uncommon features have been revealed previously in patients with TTD [1, 13, 14]. Here, we summarized the clinical presentation of our case and the published cases with TTD (Supplementary Table 3). The findings recommended that the attention to individual’s clinical presentation and comprehensive examination for multiple systems, such as regular ophthalmologic, neurology examination would be benefit to the accurate and timely diagnosis of TTD.

To date, the incidence of TTD in Asian countries is extremely low, especially in China. As far as we know, only one 3-years old girl with TDD has been reported from China [15]. The patient presented with severe TTD phenotypes, including brittle hair, developmental delay, ichthyosis, intellectual impairment, and osteosclerosis. However, no genetic evidence has been reported. Our patient also exhibited a relatively severe TTD phenotype accompanied by a compound heterozygous variants, implicating that the two gene alleles carry two different variants. The maternal variant, a deletion of 41 bp that encompasses the intron 22-exon 23 junction (c.2191-18_2213del) has been previously described by Botta, E. et al. (2009), which affects the splicing of intron 22 and generates multiple out-of-frame transcripts from codon 731 [2]. The paternal variant (c.1666-1G > A) occurs in the splice site of intron 17 located 7 bp before the intron–exon junction, likely abolishing or altering *ERCC2* gene function through aberrant splicing. The paternal variant was predicted to have a high risk of leading to a broken site and subsequently resulting in erroneous mature mRNA constitution according to in silico analysis by HSF3.0 and MaxEnt tools, which most probably affecting splicing. To the best of our knowledge, the paternal variant (c.1666-1G > A) is a novel variant so far and not reported in other cases. The two variants were all not reported in the Human Genome Variant Database (HGMD); however, using a clinical protocol for sequence variants interpretation, these two variants were classified as pathogenic, according to the American College of Medical Genetics and Genomics (ACMG) standards and ClinGen guidelines [16, 17].

So far, this is the first TTD patient with a compound heterozygous *ERCC2* variant reported from China. The finding of a pathogenic splice site variant in *ERCC2* gene expands the list of known genetic variants associated with TTD.

## Supplementary Information

**Additional file 1: Supplementary Table 1.** The output data during whole exome sequencing.

**Additional file 2: Supplementary Table 2. **The output SNP information during whole exome sequencing.

**Additional file 3: Supplementary Table 3.** Clinical presentation of our case and the published cases with trichothiodystrophy.

## Data Availability

All data generated or analysed during this study are included in this published article and its supplementary materials.

## Abbreviations

TTD: Trichothiodystrophy
HGMD: Human Genome Variant Database
ACMG: American College of Medical Genetics and Genomics

## References

[1] Pehlivan D, Cefle K, Raams A, Ozturk S, Baykal C, Kleijer WJ, Palanduz S, Jaspers NG (2012). A Turkish trichothiodystrophy patient with homozygous XPD mutation and genotype-phenotype relationship. J Dermatol.

[2] Botta E, Nardo T, Orioli D, Guglielmino R, Ricotti R, Bondanza S, Benedicenti F, Zambruno G, Stefanini M (2009). Genotype-phenotype relationships in trichothiodystrophy patients with novel splicing mutations in the XPD gene. Hum Mutat.

[3] Leemans G, De Raeve L, Keymolen K (2020). ERCC2 mutations in two siblings with a severe trichothiodystrophy phenotype. J Eur Acad Dermatol Venereol.

[4] Moriwaki S, Saruwatari H, Kanzaki T, Kanekura T, Minoshima S (2014). Trichothiodystrophy group A: a first Japanese patient with a novel homozygous nonsense mutation in the GTF2H5 gene. J Dermatol.

[5] Stefanini M, Botta E, Lanzafame M, Orioli D (2010). Trichothiodystrophy: from basic mechanisms to clinical implications. DNA Repair.

[6] Faghri S, Tamura D, Kraemer KH, Digiovanna JJ (2008). Trichothiodystrophy: a systematic review of 112 published cases characterises a wide spectrum of clinical manifestations. J Med Genet.

[7] Itin PH, Sarasin A, Pittelkow MR (2001). Trichothiodystrophy: update on the sulfur-deficient brittle hair syndromes. J Am Acad Dermatol.

[8] Tamura D, Merideth M, DiGiovanna JJ, Zhou X, Tucker MA, Goldstein AM, Brooks BP, Khan SG, Oh KS, Ueda T (2011). High-risk pregnancy and neonatal complications in the DNA repair and transcription disorder trichothiodystrophy: report of 27 affected pregnancies. Prenat Diagn.

[9] Lund EB, Stein SL (2019). Novel ERCC2 mutation in two siblings with trichothiodystrophy. Pediatr Dermatol.

[10] Usuda T, Saijo M, Tanaka K, Sato N, Uchiyama M, Kobayashi T (2011). A Japanese trichothiodystrophy patient with XPD mutations. J Hum Genet.

[11] Moslehi R, Signore C, Tamura D, Mills JL, Digiovanna JJ, Tucker MA, Troendle J, Ueda T, Boyle J, Khan SG (2010). Adverse effects of trichothiodystrophy DNA repair and transcription gene disorder on human fetal development. Clin Genet.

[12] Brooks BP, Thompson AH, Clayton JA, Chan C-C, Tamura D, Zein WM, Blain D, Hadsall C, Rowan J, Bowles KE (2011). Ocular manifestations of trichothiodystrophy. Ophthalmology.

[13] Tolmie JL, de Berker D, Dawber R, Galloway C, Gregory DW, Lehmann AR, McClure J, Pollitt RJ, Stephenson JB (1994). Syndromes associated with trichothiodystrophy. Clin Dysmorphol.

[14] Mazereeuw-Hautier J, Pech JH, Heitz F, Bonafe JL (2002). Trichothiodystrophy and congenital heart disease in two sisters. Ann Dermatol Venereol.

[15] Xu F-X, Liao X-F, Liu D-H, He Q-N (2012). One case of low sulfur hair malnutrition complicated with SIBI (D) S syndrome. Chin J Contemp Pediatr.

[16] Zhang J, Yao Y, He H, Shen J (2020). Clinical interpretation of sequence variants. Curr Protoc Hum Genet.

[17] Richards S, Aziz N, Bale S, Bick D, Das S, Gastier-Foster J, Grody WW, Hegde M, Lyon E, Spector E (2015). Standards and guidelines for the interpretation of sequence variants: a joint consensus recommendation of the American College of Medical Genetics and Genomics and the Association for Molecular Pathology. Genet Med.

