# Supplementary material for: Novel ERCC2 variant in trichothiodystrophy infant: the first case report in China
Source: BMC Pediatr. 2021 Mar 12;21:123. doi: 10.1186/s12887-021-02585-4 (PMC7955621; doi:10.1186/s12887-021-02585-4)
Supplement: Supplementary file 1 — Additional file 1: Supplementary Table 1. The output data during whole exome sequencing. [file 12887_2021_2585_MOESM1_ESM.docx]

**Supplementary Table 1.** The output data during whole exome sequencing

| NGS process | Kits |
| --- | --- |
| DNA extraction | Magnetic Beads Genomic DNA Extraction Kit (Tiagen, Beijing, China) |
| Library construction | KAPA HyperPlus Kits (Roche Diagnostics, Pleasanton, CA, USA) |
| DNA quantification | Qubit™ 1X dsDNA HS Assay Kit (Thermo Fisher Scientific, San Jose, CA, USA) |
| Capture kit | Agilent SureSelect Human All Exon V6 kit (Agilent Technologies, Santa Clara, CA, USA) |
| Sequencing platform | Illumina Hiseq 2500 sequencing platform (Illumina San Diego, CA, USA) |
| Metrics | Sequencing coverage in target region: 99%  Average sequencing depth in target region: 224  Percentage of average sequencing depth >20X in target region: 99.5% |

NGS, Next Generation Sequencing.
