# Supplementary material for: Novel ERCC2 variant in trichothiodystrophy infant: the first case report in China
Source: BMC Pediatr. 2021 Mar 12;21:123. doi: 10.1186/s12887-021-02585-4 (PMC7955621; doi:10.1186/s12887-021-02585-4)
Supplement: Supplementary file 3 — Additional file 3: Supplementary Table 3. Clinical presentation of our case and the published cases with trichothiodystrophy. [file 12887_2021_2585_MOESM3_ESM.docx]

**Supplementary Table 3**. Clinical presentation of our case and the published cases with trichothiodystrophy.

| Clinical presentation | Our case | Published cases |
| --- | --- | --- |
| Skin abnormalities | Ichthyosis; Eczema. | Ichthyosis; Eczema; Collodion membrane at birth; Photosensitivity; Freckles; Dry skin; Erythroderma; etc. |
| Hair and nail abnormalities | Brittle hair; Hair loss. | Brittle, sulphur deficient hair; Hair loss;  Onychodystrophy; Brittle nails; Hypoplasia; Koilonychia. |
| Neurologic abnormalities | Not available. | Developmental delay; Intellectual impairment; Microcephaly; Abnormal gait; Increased deep tendon reflexes; Neuroimaging abnormalities. |
| Growth abnormalities | Low weight. | Low height and/or weight; Growth retardation. |
| Sexual/reproductive abnormalities | Hypospadias. | Gonadal dysgenesis including hypogonadism, cryptorchidism panhypopituitarism, delayed pubertal development, and sexual maturation. |
| Ocular abnormalities | Retinal pigmentation. | Cataract; Retinal pigmentation; Dry eyes; Nystagmus; Strabismus. |
| Infections | Neonatal pneumonia; Recurrent infection. | Infections in the neonatal period (including respiratory, gastrointestinal, ear, skin, urinary, sepsis, etc.); Recurrent infection. |
| Skeletal and dental abnormalities | Not available. | Osteosclerosis; Delayed bone age; Osteopenia. |
| Cardiac and hepatic abnormalities | Atrial septal defect; Patent foramen ovale. | Cardiac defects (cardiomyopathy, pulmonic stenosis and ventricular septal defect); Murmur; Multiple liver haemangioendotheliomas. |
| Haematologic abnormalities | No abnormality. | Anaemia; Low mean corpuscular volume; Neutropenia;  Elevated haemoglobin A2. |
| DNA repair abnormalities and gene defects | Mutations in ERCC2. | Mutations in ERCC2, ERCC3, and GTF2H5. |
